# Whole genome sequencing of a novel *Bacillus thuringiensis* isolated from Assam soil

**DOI:** 10.1186/s12866-023-02821-0

**Published:** 2023-03-31

**Authors:** Mihir Rabha, Debajit Das, Trishna Konwar, Sumita Acharjee, Bidyut Kumar Sarmah

**Affiliations:** 1 Department of Agricultural Biotechnology, Assam Agricultural University, Jorhat-13, Assam, India; 2 Silkworm Pathology Section, Central Sericultural Research and Training Institute, Central Silk Board, Ministry of Textile, Govt of India, Berhampore, West Bengal 7421 01 India; 3 Department of Biotechnology-Northeast Centre for Agricultural Biotechnology (DBT-NECAB), Assam Agricultural University, Jorhat-13, Assam, India

**Keywords:** *Bacillus thuringiensis*, Insecticidal proteins, *Cry gene*s, Whole Genome Sequencing, *B. thuringiensis* strain BA04

## Abstract

**Background:**

*Bacillus thuringiensis* (Bt) is a gram-positive ubiquitous saprophytic bacterium that produces proteins (Crystal protein, Vegetative insecticidal protein, and Secreted insecticidal protein) toxic to insects during its growth cycle. In the present study, the whole genome of a locally isolated *B. thuringiensis* strain BA04 was sequenced to explore the genetic makeup and to identify the genes responsible to produce insecticidal proteins including the virulence factors. The strain was isolated from the soil sample of the Kaziranga National Park, Assam, North-Eastern part of India (Latitude: 26°34′39.11''N and Longitude: 93°10′16.04''E).

**Results:**

The whole genome sequencing (WGS) of the BA04 strain revealed that it has a circular genome of size 6,113,005 bp with four numbers of plasmids. A total of 6,111 genes including two novel crystal protein-encoding genes (MH753362.1 and MH753363.1) were identified. The BLASTn analysis of MH753362.1 showed 84% similarities (maximum identity) with *Cry1Ia* (KJ710646.1) gene, whereas MH753363.1 exhibited 66% identity with *Insecticidal Crystal Protein (ICP)*-*6* gene (KM053257.1). At the protein level, MH753362.1 and MH753363.1 shared 79% identity with Cry1Ia (AIW52613.1) and 40% identity with Insecticidal Crystal Protein (ICP)-6 (AJW76687.1) respectively. Three-dimensional structures of these two novel protein sequences revealed that MH753362.1 have 48% structural similarity with Cry8ea1 protein, whereas MH753363.1 showed only 20% structural similarity with Cry4Aa protein.

Apart from these insecticidal genes, the strain was also found to contain virulence and virulence-associated factors including the antibiotic resistance genes and Clustered regularly interspaced short palindromic repeat (CRISPR) sequences.

**Conclusion:**

This is the first report on the whole genome sequence of Bt strain BA04 isolated from Assam, a North-Eastern state of India. The WGS of strain BA04 unveils the presence of two novel types of insecticidal crystal protein-encoding genes which can be used for the development of insect-resistant transgenic crops. Additionally, the strain could be used for the formulations of effective biopesticides. The WGS provides the fastest and cheapest platform for a better understanding of the genetic makeup of a strain and helps to explore the role of virulence genes in pathogenicity against the insect host.

**Supplementary Information:**

The online version contains supplementary material available at 10.1186/s12866-023-02821-0.

## Background

*Bacillus thuringiensis* is a gram-positive, saprophytic, spore-forming, entomopathogenic bacteria that can potentially be used as a biocontrol agent. *B. thuringiensis* produces crystal proteins (delta endotoxins) during sporulation which confers toxicity against insect pests [1–3]. These crystal proteins are generally classified into different groups and sub-groups based on their amino acid sequence similarities and the specificity of toxicity against insect pests of different orders such as lepidopteran, coleopteran, dipteran, homopteran, hymenopteran, Mallophaga, and other organisms such as nematodes, protozoa and mites [4–6]. Due to the specificity of these crystal proteins, *B. thuringiensis* is one of the most widely studied biocontrol agent at the molecular level. These crystal proteins are encoded by *Cry* genes and to date, more than 800 *Cry* gene sequences have been identified from different *B. thuringiensis* strains and registered on the NCBI site [7]. Despite all these, the mining of novel *Cry gene*s is remaining an interesting area of research to explore novel candidate genes having higher efficacy and toxicity against a wide range of insect pests.

Several molecular approaches have been adopted in the recent past to search for a novel type of *Cry* genes such as gene hybridization [8, 9], PCR-mediated techniques by using general or multi-primer [10], DNA library [11], and PCR followed by restriction fragment analysis [12]. Howbeit, all these techniques are labor-intensive, time-consuming, and inefficient compared to the whole genome sequencing approach.

Apart from these insecticidal crystal proteins, strains of *B. thuringiensis* are known to produce other insecticidal proteins such as Vegetative Insecticidal Protein (VIP), Secreted Insecticidal Protein (Sip), and Cytotoxic proteins (Cyt) at different stages of its growth cycle. Thus, Bt strains are highly copious of insecticidal genes which might help them to survive and proliferate in different ecological and geographical conditions. The whole genome sequence analysis of *B. thuringiensis* from different geographical locations and ecological habitats would help us to understand the integrity of their genome and the precise order of evolutions. The increasingly available online resources, databases, and archives of the WGS data along with the parallel progress in the field of bioinformatics have remarkably reduced the cost of genome sequencing. The large-scale availability of WGS data has facilitated the identification, characterization, and mapping of genomes of new organisms and detailed metabolic pathways of such organisms, fishing out new candidate genes having broad spectrum activity against insects and comparing genomes across multiple samples. The whole genome sequencing of *B. thuringiensis* provides an opportunity to investigate in detail the genetic makeup for pathogenicity and toxicity against the host insects. In the present investigation, we identified and isolated novel types of insecticidal crystal protein genes from a *B. thuringiensis* strain of Assam soil. Since the diversity in the ecological habitat and geographical locations plays a vital role in finding variations in the *Cry* genes of *B. thuringiensis* [13, 14]. Assam is a bio-diversity hotspot endowed with a variety of natural flora and fauna, making it a unique natural habitat for novel *B. thuringiensis* strains with novel insecticidal genes. For the present investigation, the Bt strain was isolated from the soil samples of Assam, and we performed morphological, biochemical, and molecular characterization to identify novel genes [15, 16].

From the taxonomical classification point of view, *B. thuringiensis* belongs to the *B. cereus* group that consists of six species (*B. thuringiensis*, *B. anthracis*, *B. cereus*, *B. mycoides*, *B. pseudomycoides* and *B. weihenstephanensis*) [17]. Based on the previous reports, species of *the B. cereus* group share a very high level of protein homology and show similar orientations of conserved sequences in the genome [18]. Out of which, three species *B.thuringiensis*, *B. cereus*, and *B. anthracis* were found to have similar genetic makeup [19, 20]. However, among these three species, *B*. *cereus* and *B. thuringiensis* are considered closely related species with almost similar kind of genetic makeup. *B. cereus* is considered acrystalliferous, whereas *B. thruinigensisis* is known as crystalliferous bacteria that produces crystal proteins. This is the only property that differentiates these two species. In case of loss of these crystal protein-encoding genes located on the plasmid (transfer through conjugation), it becomes nearly impossible to differentiate them. The previous report also concluded that these two species cannot be separated based on phylogenetic analysis [17]. Therefore, a whole genome study can help to understand their genetic makeup to differentiate these two closely related species more effectively.

## Results

### Insecticidal activity of the strain BA04

Larvae of *Helicoverpa armigera* of various instars raised on artificial diet were fed with spores of the *B. thuringiensis* strain BA04. After 7 days, considerable (98–100%) mortality of larvae was observed irrespective of the larval instars (Supplementary Fig. 2).

### Genome sequencing and assembly of *B. thuringiensis* strain BA04

A total of 9,293,122 reads were generated after filtering with a quality score of 94.89% and as many as 94 contigs were obtained. The total size of the genome was found to be 6,113,005 bp with an average GC% of 34.78164 (Table 1). The sequencing depth was 153X [Seq. depth = 938,605,322 no. of bases obtained /6113005 bp size of the genome = 153.54] and the coverage was 99.99% [ Coverage (%) = (65,031 read length × 94 No. of reads)/ 6,113,005 genome length × 100]. The genome of the BA04 strain of Bt was comprised of 4 numbers of plasmids.

**Table 1:** Genomic features of B. thuringiensis isolate BA04

Genome features	Value
Number of contigs	94
Genome size (bp)	6,113,005
GC percent	34.78164
Number of plasmid	4
Total genes	6111
Protein coding genes	6038
tRNA	65
rRNA	7
tmRNA	1

*-Bases (bp) *The number of bases in each contig.

*-Gene *The basic physical unit of heredity,

*-CDS *Coding Sequence

*-tRNA *Transfer RNA,

*-rRNA *Ribosomal RNA, a molecular component of ribosome.

*-tmRNA *Transfer-messenger RNA (dual tRNA-like and messenger RNA-like properties).

### Genome annotation

**Fig. 1:**
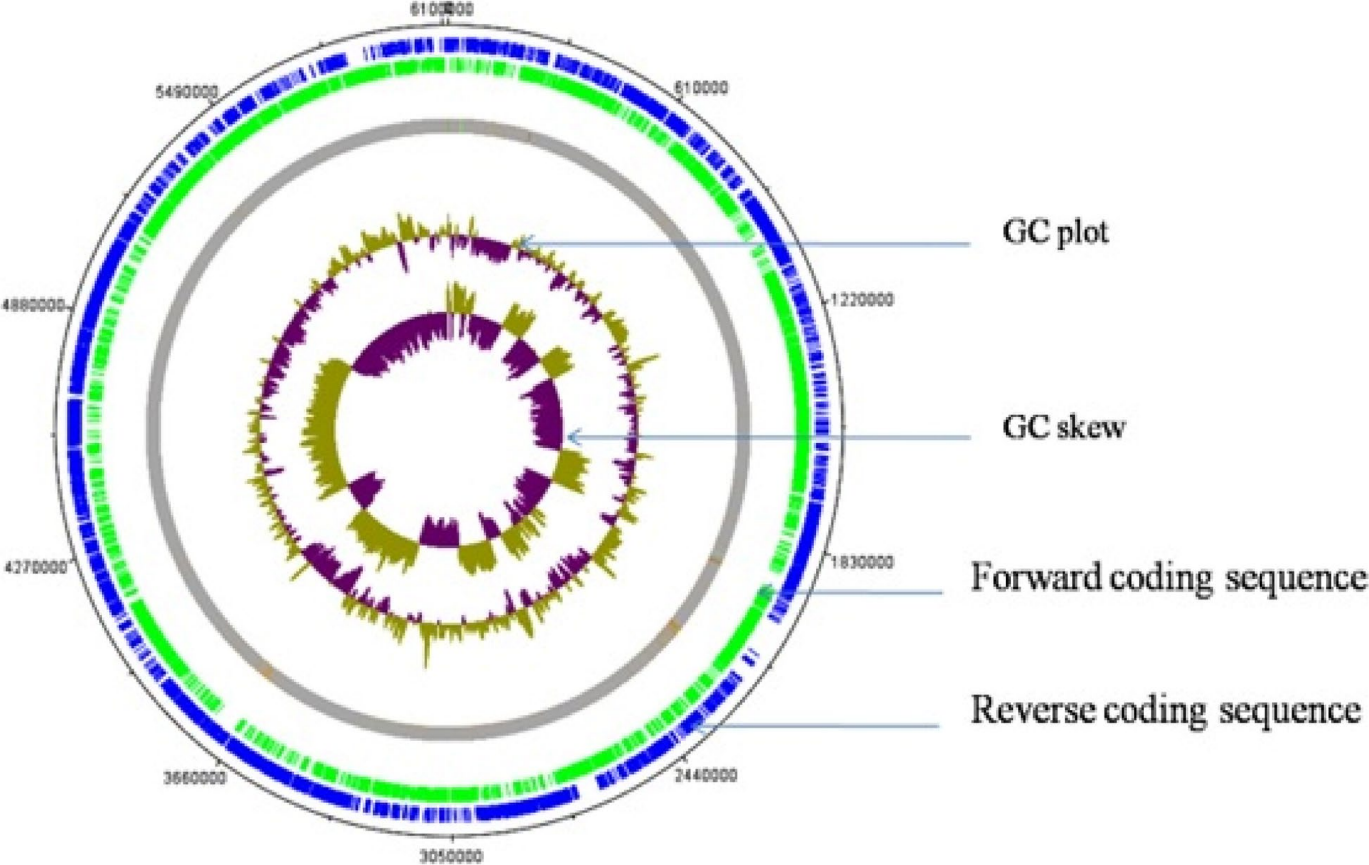
Circular genome map of *Bacillus thuringiensis* isolate BA04 generated by DNA plotter tool

Genome annotation revealed the presence of 6111 genes corresponding to a total of 6038 protein-encoding genes (98.80% of all the genes). The non-coding RNAs include 67 tRNA (~ 1.1% of all the genes), 7 rRNA genes (0.11% of all the genes), and one tmRNA (Transfer-messenger RNA), which is a bacterial RNA molecule with dual tRNA-like and messenger RNA-like properties (Table 1). The tmRNA was known to play a major role in the ribosome rescue process and quality protein synthesis [21]. The circular genome obtained through the DNA plotters showed the quality of the genome sequence (both forward and reverse sequences). Figure 1 has both forward sequences (blue circle) and reverse sequences (green circle) where the GC content and GC skew are also flaunted. The circular form of the whole genome of strain BA04 has been obtained using the DNA plotter tool available at http://www.sanger.ac.uk/science/tools/dnaplotter. DNA plotter produces the genome in a linear and circular form where the quality of the sequences can be observed in a graphical format [22].

**Fig. 2:**
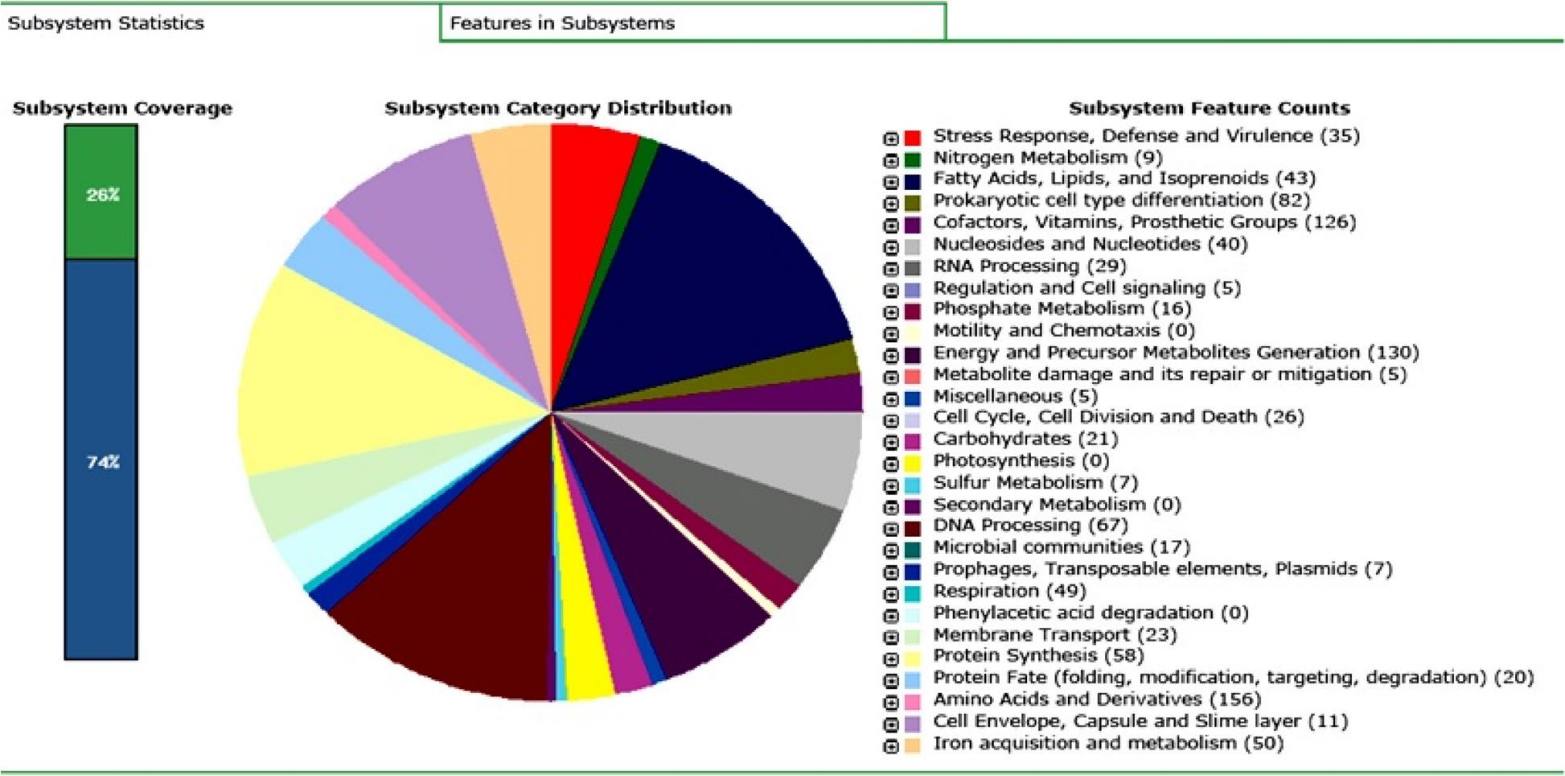
The RAST analysis-based subsystem distribution of whole genome sequence of isolates BA04. Each colour in the pi graph represents a particular group of genes mentioned in the right site of the graph

The Rapid Annotations using Subsystems Technology (RAST) annotation have distributed 6283 genes into 180 subsystems. The subsystems of the BA04 strain genome based on the RAST annotation server are depicted in Fig. 2. The most abundant genes annotated were associated with amino acids synthesis and their derivatives (156 genes; 2.48%) followed by energy and precursor metabolites generation (130 genes; 2.07%) and then cofactors, vitamins, and prosthetic groups (126 genes; 2.00%). The sketch of the KEGG (Kyoto Encyclopaedia of Genes and Genomes) metabolic pathway obtained through the RAST analysis was provided in the supplementary data (Additional file 1: Figure S1) and with the list of all the genes identified through the RAST server (Additional file 2: Table S1).

### Comparison with closely related *B. thuringiensis* strains

**Fig. 3:**
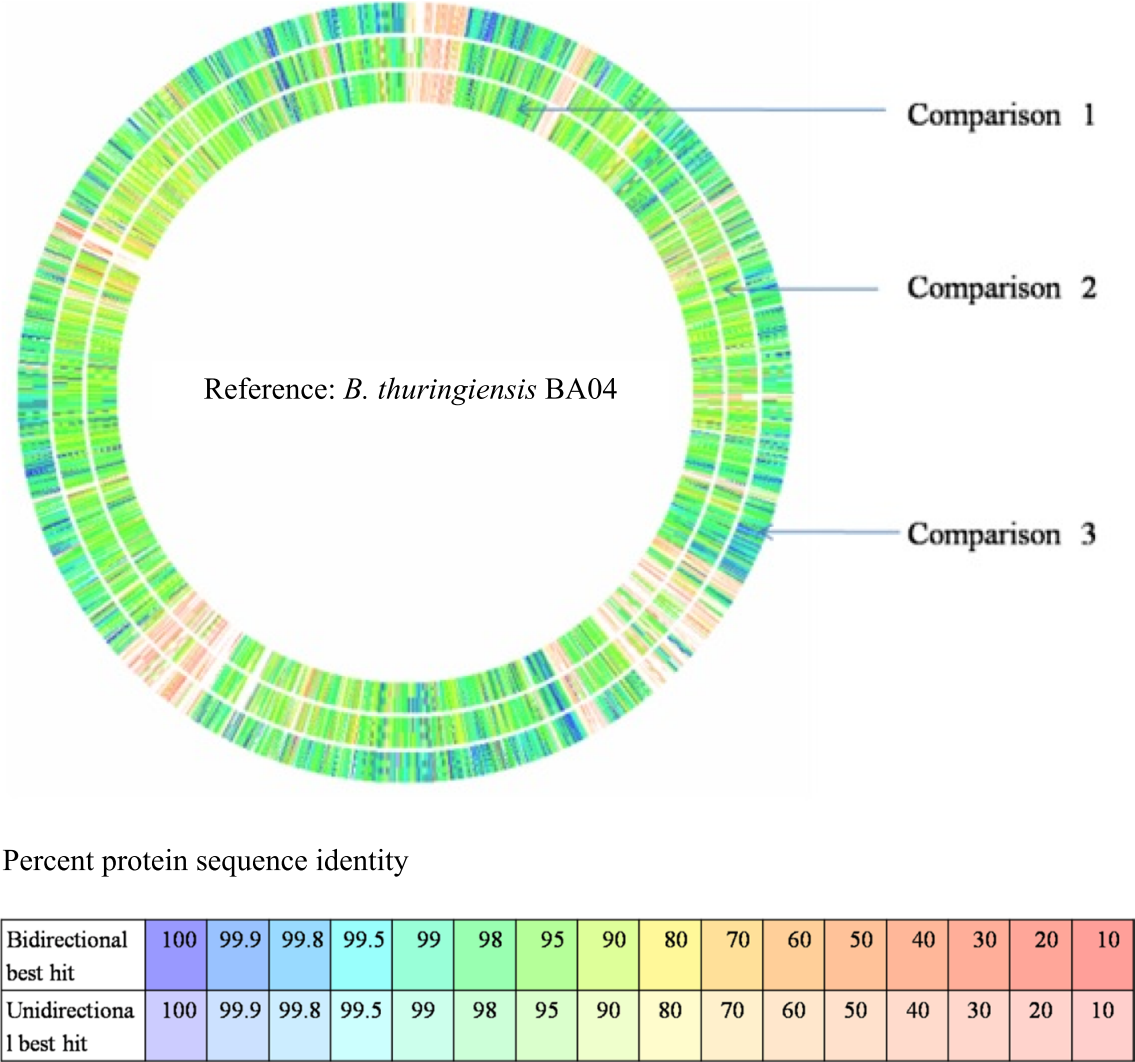
Comparative genome analysis of isolate BA04 under RAST server. The first circle Comparison 1 is with *B. cereus* strain ATCC 14,579. The second circle Comparison 2 is with *B. cereus* biovar *anthracis* str. CI (637,380.6) and the third circle Comparison 3 is with *B. thuringiensis* serovar konkukian str. 97–27 (281,309.3)

A comparative protein-encoding genome sequence analysis of BA04 strain with two *B. cereus* genomes (*B. cereus* ATCC 14,579 and *B. cereus* biovar *anthracis* str. CI) and a different sub-species of *B. thuringiensis* (*B. thuringiensis* serovar konkukian str. 97–27) revealed that BA04 genome has a significant variation with the *B. cereus* strains (Fig. 3). Interestingly, noticeable variations were also found between BA04 and *B. thuringiensis* serovar konkukian strain 97–27. The whole genome BLAST analysis of BA04 has shown close relation with another *B. thuringiensis* strains HS18-1 with 99% identity (82% query cover and maximum alignment score 4021 with 0.0 error value) rather than with *B. thuringiensis* serovar konkukian str. 97–27 (64.8% similarity). Also, BA04 showed 73.6 and 63.8% similarity with *B. cereus* ATCC 14,579 and B. cereus biovar anthracis strain, respectively.

### Phylogenetic assessments

**Fig. 4:**
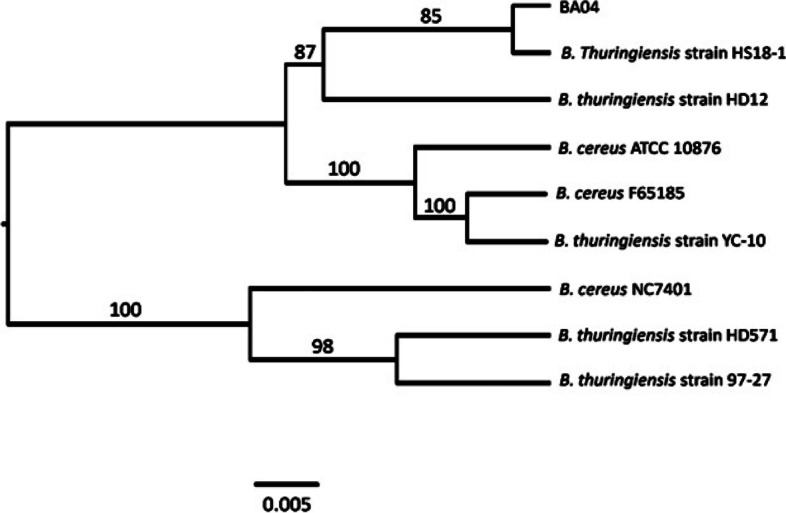
Phylogenetic tree obtained using the Genome-to-Genome Distance Calculator 2.1 (GGDC)

The Genome-to-genome distance calculator (GGDC) analyses indicated that the strain BA04 is closely clustered with *B. thuringiensis* strain HS18-1 (with 92.3% similarity) followed by *B. thuringiensis* strain HD12 (71.5% similarity). The distance of the tree was about 0.005. However, the *B. cereus* strain showed a distant relation with strain BA04 and clustered in separate lineages (Fig. 4). The numbers above the branches are greedy-with-trimming pseudo-bootstrap support values above 80% are shown. *thuringiensis.*

### Genomic islands (GIs) of *B. thuringiensis* strain BA04

**Fig. 5:**
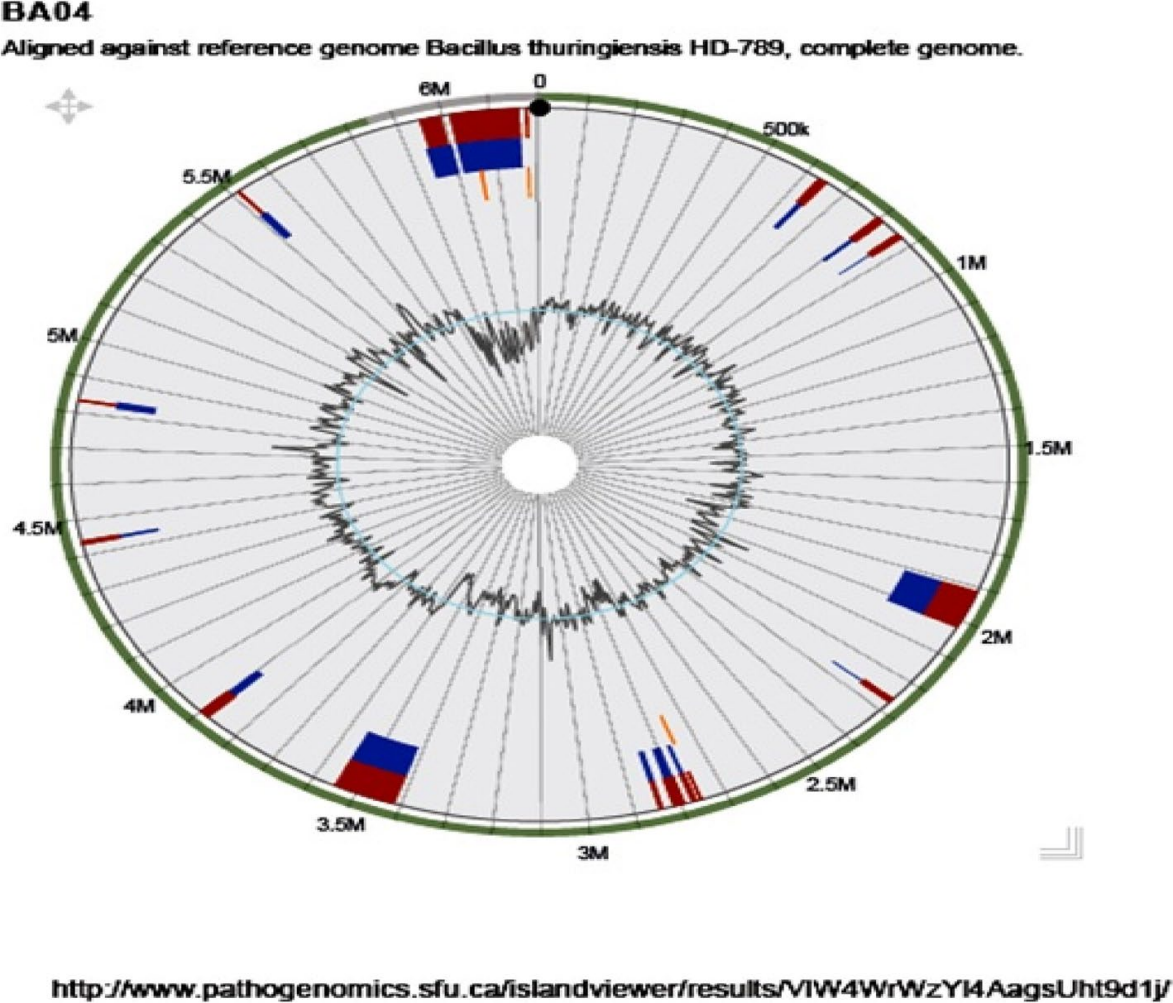
Genomic Islands prediction and genome visualization of isolates BA04 against Bt strain HD789 revealed 17 genomic islands

A total of 17 genomic islands mostly containing genes of unknown function i.e. hypothetical proteins (222) with 191 clustered genes have been identified in the genome when compared with the reference genome of *B. thuringiensis* strain HD789 using the Island Viewer 4 (http://www.pathogenomics.sfu.ca/islandviewer/browse/) (Fig. 5). Moreover, genes encoding putative transposase (4), PD-(D/E)XK nuclease family transposase (2), putative prophage phiRv2 integrase (1) were also represented in the GIs. These genomic islands are the regions of the genome that provide evidence about the horizontal gene transfer which play important role in the evolution, and diversification of pathogenic microbes and the adaptation of bacteria to different environments [23]. The list of the genes that were clustered in the genomic islands and their locus in the genome are included as supplementary data (Additional file 3: Table S2).

### Identification of two novel *Cry* genes

**Fig. 6:**
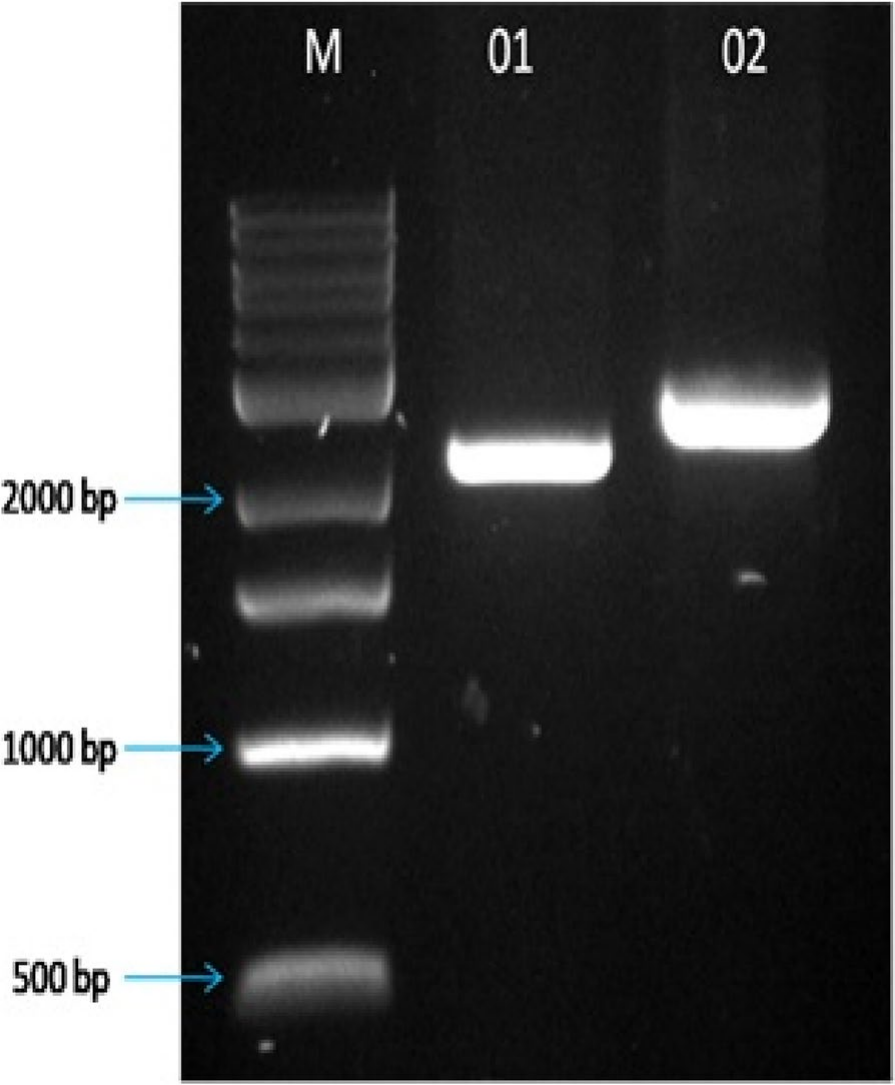
Amplification of full length identified *Cry* gene sequences from isolate BA04. Lane M- 1 kb DNA ladder; Lane 1: amplified product of MH753362.1; Lane 2: Amplified product of MH753363.1. The gel image was cropped to show the desired amplicons. Original gel image was included as additional figure (Additional file 1_Figure S1)

The genome annotations of the strain BA04 have led to the identification of two novel types of *Cry* genes which were deposited at the NCBI (MH753362.1 and MH753363.1), the size of MH753362.1 and MH753363.1were found to be 2.1 and 2.7 kb, respectively. Full-length primers were designed for both genes and were successfully PCR amplified (Fig. 6). Subsequently, the purified PCR products were cloned into a pGEM®-T easy cloning vector for further analysis. The BLASTn analysis revealed that MH753362.1 has a maximum sequence similarity of about 84% with *Cry1Ia* (KJ710646.1), while MH753363.1 showed 66% identity with the *ICP-6* gene (KM053257.1). The pBlast of MH753362.1 showed a maximum of 79% identity with Cry1Ia (AIW52613.1) like protein, and MH753363.1 exhibited 40% identity with ICP-6 (ID AJW76687.1). These results indicated significant variations in the sequences of both MH753362.1 and MH753363.1 and could be considered novel types of crystal protein-encoding genes, however, the toxicity assay through expression analysis and insect bioassay could add to their efficacy towards target insects.

**Fig. 7:**
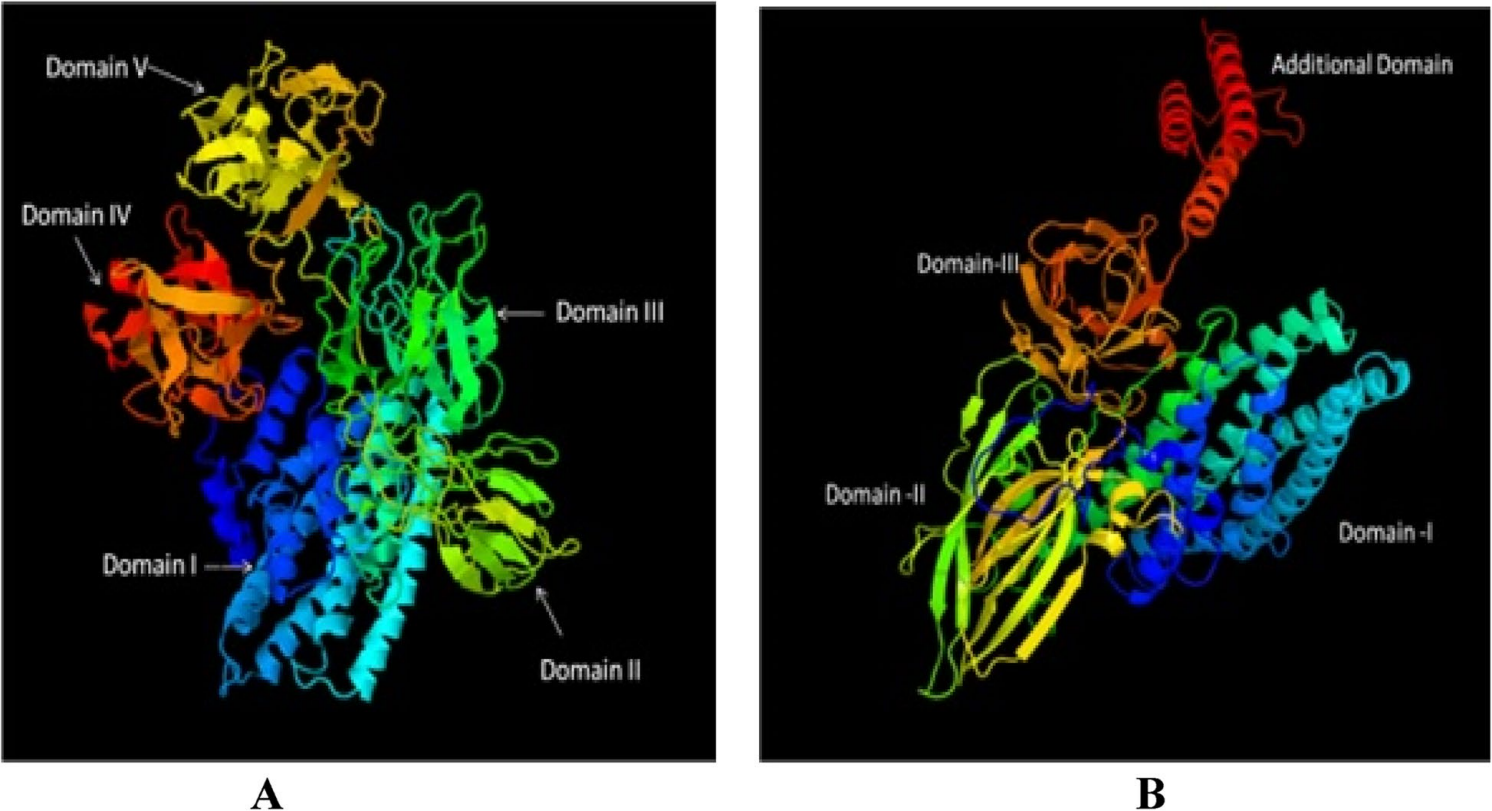
Three-D analysis for Cry protein like sequences under online server Phyre2. (A) 3D structure obtained from sequence MH753362.1, (B) 3D structure obtained from sequence MH753363.1

Analysis of the three-dimensional structures of these two novel proteins using the online server Phyre2 revealed 48% structural identities with Cry8Ea1 for MH753362.1, whereas MH753363.1 was only 20% similar to Cry4Aa proteins. The 3D analysis of both sequences denoted the presence of three functional domains of a typical crystal protein along with a few additional domains (Fig. 7).

### Genome properties

The genome of *B. thuringiensis* strain BA04 also has many virulence factors (Table 2) apart from the Cry toxins. During the genome annotation, two different types of chitinases (*chiA* and *chiD*) along with different types of protease encoding genes including four bacillolysin, five collagenase, four different types of immune inhibitors, and three phospholipase encoding genes were identified (Table 2). Three different lactonase encoding genes, alveolysin, zeta toxin (*Streptococcus pyogenes*), and toxin A were also found in BA04. Also, different types of hemolysin-encoding genes were identified during the genome annotation (Table 2). We also found numerous virulence-associated factors (Table 3) including antitoxin gene, extracellular metalloprotease, spore photoproduct lyase, capsule biosynthesis genes, sporulation protein genes, spore germination protein genes, and genes encoding three different potential bacteriocins such *asalbA, albE*, and *albF* (Table 3). The strain was also found to carry genes associated with nitrogen metabolism (Table 3). In all, 8 nitrogen metabolism-related genes were identified corresponding to *ureA, ureB, ureC, ureD, ureE, ureF, ureG, ureH*, and *ureI* which play a crucial role in the synthesis of urease. Furthermore, the genome of the BA04 strain was found to encode several antibiotic resistance genes such as vancomycin, tetracycline, polymyxin, bicyclomycin, fosfomycin, polymyxin, and fosmidomycin resistance genes (Table 4). We identified 11 genes associated with multidrug resistance genes of which 5 genes are related to ABC transporter. The strain was also found to contain five different types of CRISPR sequences (Table 5).

**Table 2:** A list of virulence factors encoding genes other than Cry toxin of B. thuringiensis isolate BA04

Virulence factor	Number	Locus tag
Bacteriocin	3	
albE		s15_02010
albF		s15_02009
albA		s15_06070
Proteases		
Bacillolysin	4	
npr1		s15_00620
npr_2		s15_00919
npr_3		s15_04094
nprM		s15_05144
Collagenases	5	
colA_1		s15_00088
colA_2		s15_01985
colA_3		s15_03845
colA_4		s15_03865
colA_5		s15_05435
Immune inhibitor	4	
ina_1		s15_01231
ina_2		s15_04166
ina_3		s15_04315
ina_4		s15_05414
Phospholipase	3	
plc_1		s15_02179
ytpA		s15_03179
plc_2		s15_04172
Chitinases	3	
chiA1_1		s15_00514
chiA1_2		s15_01673
chid		s15_01944
N-acyl homoserine lactonase	3	
attM_1		s15_00124
attM_2		s15_00836
aiiA		s15_01744
Enterotoxin	1	
hblA_1		s15_00184
Hemolysin A	1	
tlyA		s15_02850
Hemolysin C	1	
tlyC		s15_01423
Hemolysin BL	6	
hblA_1		s15_00185
hblA_2		s15_00186
hblA_3		s15_01601
hblA_4		s15_01602
hblA_5		s15_02546
hblA_6		s15_02547
Hemolysin II	1	
Hly		s15_03857
Gamma hemolysin	1	
hlgB		s15_03550
Alveolysin	1	
Alv		s15_05712
Zeta toxin	1	s15_00623
Toxin A	1	
toxA		s15_03685

**Table 3:** List of genes encoding virulence associated factors of *B. thuringiensis* isolate BA04

Genes	Numbers	Locus tag
**Antitoxin protein**	4	
ndoAI		s15_00663
yokJ		s15_05447
hipB		s15_05774
yezG		s15_01132
**Spore photoproduct lyase**	1	
splG		s15_02820
**Extracellular metalloprotease precursor**	1	
mpr		s15_02435
**Putative metalloprotease**	1	
ypwA		s15_04590
**ATP-dependent zinc metalloprotease**	1	
ftsH		s15_01911
**Genes involved in nitrogen metabolism**	8	
ureA (gamma)		s15_03980
ureB (beta)		s15_03979
ureC (alpha subunit)		s15_03978
ureD		s15_03974
ureE		s15_03977
ureF		s15_03976
ureG		s15_03975
ureI_1		s15_03973
**Capsule biosynthesis protein**	2	
CapA		s15_01142
CapA_2		s15_01629
**Stage V sporulation protein T**	2	
spoVT		s15_01898
spoVS_2		s15_01999
**Stage II sporulation protein E**	2	
spoIIE		s15_01908
spoIIGA		s15_02128
**Spore germination protein**	5	
gerD		s15_02579
yndE		s15_02226
gerBA_2		s15_02225
gerAA_2		s15_03155
gerPF_2		s15_03223

**Table 4:** A list of antibiotic resistance genes identified in B. thuringiensis isolate BA04

**Antibiotic resistane gene**	**Numbers**	Locus tag
**Multidrug resistance**	19	
mdtH_1		s15_00672
mdtH_2		s15_01211
mdtH_3		s15_02501
mdtH_4		s15_03959
mdtH_5		s15_05299
stp_1		s15_01506
norM_1		s15_03626
norM_2		s15_04412
ykkC		s15_02811
ykkD_1		s15_02494
ykkD_2		s15_02810
bmr3_1		s15_00395
bmr3_2		s15_03132
bmr3_3		s15_04158
bmr3_4		s15_05294
bmrA_1		s15_01552
bmrA_2		s15_02657
emrK		s15_02986
emrY		s15_01657
**Vancomycin B-type resistance**	3	
vanW_1		s15_03872
vanW_2		s15_04098
vanW_3		s15_05415
**Putative multidrug resistance ABC**	5	
yheH_1		s15_02508
yheH_2		s15_04023
yheI_1		s15_02507
yheI_2		s15_02528
yheI_3		s15_04024
**Tetracycline resistance protein, class C**	6	
tetA_1		s15_00274
tetA_2		s15_01626
tetA_3		s15_02496
tetA_4		s15_04169
tetA_5		s15_04827
tetO		s15_05454
**Daunorubicin/doxorubicin resistance ATP-binding protein**	6	
drrA_1		s15_01763
drrA_2		s15_02952
drrA_3		s15_04767
drrA_4		s15_04937
drrA_5		s15_05968
drrB		s15_01764
**Tellurite resistance protein**	1	
TehB		s15_04950
**fosfomycin resistance protein**	1	
FosB		s15_05424
**Multiple antibiotic resistance protein**	2	
marA		s15_05470
marR		s15_01821
**Bifunctional polymyxin resistance protein**	1	
arnA		s15_00573
**Bicyclomycin resistance protein**	1	
bcr		s15_00728
**Fosmidomycin resistance protein**	1	
Fsr		s15_01651

**Table 5:** Details of CRISPR sequences of BA04 genome obtained through the CRISPRfinder

CRISPR candidate	CRISPR start position	CRISPR end position	CRISPR length	Direct Repeat consensus sequences	DR length	No. Of Spacers
1	615,849	615,958	109	TACGTAGATAAAGAAGGAAAAGAAATCGCACAACGCAATA	40	1
2	2,288,456	2,288,577	121	TGAACGCCCTGAGGTCCAGTAGG	23	2
3	4,184,871	4,184,944	73	CATCACCATGGAGGACACAATCA	23	1
4	4,622,492	4,622,808	316	TTTCTTAGCTTCTTCTTCAGCTTTTTTCT	29	5
5	5,727,442	5,727,519	77	CGCTGGAGCTGGATATAACTAAAAG	25	1

*CRISPR* Clustered Regularly Interspaced Short Palindromic Repeats, *DR* Direct repeats

## Discussion

In the present study, the whole genome sequencing of a new *B. thuringiensis* strain BA04 isolated from Assam soil was carried out. The WGS revealed that the genome of the BA04 strain is substantially large. It is widely documented that the strains with large genome sizes have higher toxicity than that of smaller genomes against the target pest [18] since the larger genomes contain a high copy number of plasmids having insecticidal activity. Moreover, the strains with higher toxicity generally produce different types of virulence factors which enhance the toxicity of the insecticidal genes [24]. The virulence genes are mostly reported to be involved either in adhesion, recognition or degradation that increases the pathogenicity of the bacteria [18]. These virulence factors help to invade the host atrociously, for example, chitinases are involved in the degradation of chitin present in exoskeletons of insects, bacillolysin (Zinc metal endopeptidase) which performs the hydrolysis of the amino leucine and phenylalanine that elicit the innate immune system [25]. Collagenases and phospholipases were reported to be associated with the disruption of the intestine and midgut epithelial cells that help the pathogen to colonize inside the host [26, 27]. Additionally, the strains were found to carry different types of immune inhibitors (4 genes) which are metalloprotease causing the degradation of antibacterial proteins produced by the host insect and helping the bacteria to invade the host cells [28]. Capsule biosynthetic genes plays role in the synthesis of the polymeric capsule that protects it from the pathogen [29]. In *B. anthracis*, capA, capB, and capC proteins are linked to the synthesis of polyglutamic acid capsules which are vital for host invasion [30].

Most bacteria produce bacteriocins that have broad-spectrum antimicrobial properties against viruses, fungi, and cancer cells [31] that help the bacteria to survive and compete with other microbes. BA04 strain possesses three genes encoding for bacteriocins which might have similar roles to play. The bacterial strains were also reported to carry sequences for Zeta toxins, which were known to cause toxicity against the gram-positive and gram-negative bacteria and were reported to involve in programmed cell death in bacteria [32, 33]. Zwittermycin (ZmA) another important antimicrobial protein identified from the strain found to enhance the toxicity of the crystal protein against the host insects. Also, the above antimicrobial genes and numerous multi-drug resistance genes were identified in bacteria which depict that the strain can survive extreme exposure to multiple antibiotics.

The WGS also revealed a cluster of urease synthase genes in the BA04 strain. Previous studies have demonstrated that these genes are involved in the recycling of environmental nitrogen and act as a virulence factor in pathogenic microorganisms associated with gastric ulceration and urinary stone formation [34]. The urease gene family creates favorable conditions for the bacterial pathogen by neutralizing the gastric acid in the guts of the insect host and helping the bacterial pathogen to use the ammonia for protein synthesis [34]. The insecticidal activity of *B. thuringiensis* is generally attributed to the production of compounds such as chitinase that degrade chitin. These insecticidal chitinases are mostly involved in the perforation of the peritrophic membrane that helps the microbes and their toxins to enter inside the peritrophic membrane of the host insect, thereby enhancing the activity of membrane binding toxins which increases the virulence of the pathogen [35, 36].

A recent toxin gene co-occurrence network study has revealed that different strains of *B. thuringiensis* are capable of accumulating multiple toxins with similar targets in a single cell [37], which serves as one of the most powerful strategies for delaying the development of host resistance. All these clusters of information unveil the complexity and the use of different pathways to cause toxicity against the insect host by different *B. thuringiensis* strains. Hence, the information generated through the WGS of *B. thuringiensis* strain BA04 would be helpful to understand the underlying mechanisms of these pathways. Furthermore, WGS would also facilitate comparative genomic studies to elucidate microbial evolutionary relationships.

The whole genome sequencing has revealed that a single *B. thuringiensis* strain may contain more than one type of crystal protein-encoding gene. Previously the Bt strain HD-1 was found to contain six crystal protein-encoding genes (viz. *Cry1Aa*, *Cry1Ab*, *Cry1Ac*, *Cry1Ia*, *Cry2Aa*, and *Cry2Ab*) [18]. In the present investigation, we identified two new types of *Cry* genes MH753362.1 and MH753363are similar to *Cry1Ia* (KJ710646.1) and *ICP-6-*like gene (KM053257.1) respectively. However, both these sequences have significant variations which suggest that these are novel crystal protein-encoding genes. Previous studies have shown that proteins grouped in Cry1I are effective against insects belonging to Plutellidae, Chrysomelidae, Tortricidae, Noctuidae families, etc. [38]. However, the precise mode of action of ICP-6-like proteins is yet to be unknown.

The 3D analysis showed that apart from the normal active three domains (Domain-I, Domain-II, and Domain-III) of Cry protein, both the sequences have a few additional domains that could be part of the protoxin or non-truncated protein. Based on the previous reports, domain-I having a cluster of seven α-helices is responsible for pore formation in the membrane [39, 40], whereas domain-II is composed of three anti-parallel β-sheets and domain-III has a β-sandwich of two antiparallel β-sheets involved in receptor recognition and binding [41–45]. In most cases, the activated toxin binds to receptor molecules of midgut epithelial cells and forms non-selective pores near the vicinity of the receptor binding site that cause the lysis of cells and finally kill the insects [46, 47]. The sequence MH753362.1 had a total of four numbers of domains (Fig. 7A) which includes one additional domain which is similar to that of the protoxin of Cry1Ac [39]. Likewise, the sequence MH753363.1 showed two additional domains besides the active three-domain structure (Fig. 7B). In the present study, the 3D analysis of both MH753362.1 and MH753363.1 genes were found to have similar structural domains.

The phylogenetic tree obtained through the genome-to-genome distance comparison (GGDC) tool placed the BA04 strain close to *B. thuringiensis* strain HS18-1. This strain isolated from the Sichuan basin of China was found to confer high toxicity against both lepidopteran and dipteran insects. The strain contained a few potential insecticidal genes (*Cry30Ga*, *Cry4Cb1*, *Cry50Aa1*, *Cry69Ab1*, *Cry30Ea*, *Cry54Ba*, *Cry70Aa*, *Cry71Aa*, *Cry72Aa*, and *Cry56Aa*) based on the WGS [20]. The WGS provided information about the genetic makeup of BA04 and the possibility of using it as a biocontrol agent against target insect pests. Therefore, knowing the genomic evolution of BA04 was pertinent.

The genomic islands (GIs) represent mostly the group of genes related to the horizontal origin that involves genetic exchange in bacteria and the archaeal genome [23]. GIs play a major role in genomic evolution and adaptation in a particular habitat. The Island viewer 4 is a tool based on a comparative genomics approach that helps to identify the genomic islands. The genes which are found in GIs of BA04 are mostly responsible for encoding virulence factors, antimicrobial compounds, and metal resistance genes that are effective against pathogen outbreaks [48–50].

In the present investigation, the CRISPR finder (http://crispr.i2bc.paris-saclay.fr/Server/) is used to identify the CRISPR sequences. CRISPR finder is an efficient tool as it allows the identification of the CRISPR sequences and their characteristics with their precise locations in the genome [51]. CRISPR elements play a major role in the bacterial immune system which helps to eliminate foreign genetic materials [52]. The presence of CRISPR elements in the BA04 strain confirmed the role of these sequences to resist any exogenous DNA of bacteriophages.

The whole genome sequencing of strain BA04 has made it easy to understand the genetic makeup along with the identification of two new insecticidal genes with other virulence factors. The availability of these genome sequences in the database will further help in genome annotation and evolutionary studies.

## Conclusion

Whole genome sequencing is a rapid way to characterize a microbe that can explore the genetic makeup with accuracy. Databases like NCBI, DDBJ, and EMBL played a crucial role in annotating the whole genome sequences of BA04. The whole genome sequence analysis of our local strain BA04 helped to generate information on two novel types of *Cry* genes which can be further used for the development of bioformulations and insect-resistant transgenic crops. The availability of the WGS of BA04 from India contributed to enriching the Bt database and could be used as a reference strain for the characterization of the Bt strain from NE of India in the future.

## Methods

### Bioassay

BA04 was cultured and incubated at 37℃ for 72 h, then spores were collected and washed with sterile water and diluted to an OD of 0.5 to 0.6. The artificial diets of size 1 cm squares were dipped into the spore solution and feed to various instars of larvae of *Helicoverpa armigera* as described by El-kersh and co-workers in 2016 [53]. The mortality data were recorded after one week.

### Growth condition and genomic DNA isolation

The total DNA was isolated according to the protocol mentioned by Sullivan and Klaenhammer [54] with slight modification. The culture was incubated at 37ºC (120 rpm) overnight in 100 ml of T3 medium [55] and the pallet was collected through centrifugation at 6000xg in an Eppendorf tube. The pallet was resuspended in 200 µl of lysozyme buffer (25% sucrose, 30 mg/ml lysozyme) and incubated at 37 ºC for 30 min., then 400 µl of SDS buffer (1% SDS, 0.2N NaOH) was added to the mixed solution and incubated for 7 min. Then 300 µl of ice-cold sodium acetate (pH-4.8) was mixed thoroughly and incubated on ice for 5 min. The lysate was centrifuged at 12000xg (4ºC) for 15 min and the supernatant was collected and treated with phenol three times. The DNA was precipitated with two volumes of ethanol followed by centrifugation at 13000xg for 15 min at 4ºC. The pallet was washed with 70% alcohol, dried, and dissolved in 40 µl of Tris–EDTA (Tris–CL; 10 mM and EDTA; 1 mM) buffer and the quality was observed under 0.8% agarose gel.

### Library construction

High-quality DNA was used for constructing the library with the help of the TruSeq Nano DNA kit (Cat. No. FC-121–4001). The sequencing library is prepared by random fragmentation of the DNA sample followed by 5' and 3' adapter ligation. Alternatively, "tagmentation" combines the fragmentation and ligation reactions into a single step which greatly increases the efficiency of the library preparation process. Adapter-ligated fragments are then PCR amplified and gel purified. The purified products were subjected to sequencing.

### Sequencing and annotation

The whole genome sequencing (WGS) was done on the Illumina SBS (sequencing by synthesis) platform. This sequencing technology utilizes a proprietary reversible terminator-based method that detects single bases as they are incorporated into DNA template strands. As all 4 reversible, terminator-bound dNTPs are present during each sequencing cycle, natural competition minimizes incorporation bias and greatly reduces raw error rates compared to other technologies. The result is highly accurate base-by-base sequencing that virtually eliminates sequence-context-specific errors, even within repetitive sequence regions and homopolymers. The quality of the raw data (sequences) were analyzed under Fast QC which allows for performing simple quality control checks on raw sequence data obtained from high throughput sequencing. The sequence reads were filtered before assembly so that for a pair of PE (Pair End) reads, each read has more than 90% of bases with base quality greater than or equal to Q20. The value of K-mer was analyzed using the JELLYFISH. These K-mers are the sequences of length K that were obtained during the sequencing of DNA. The De-novo assembly was done on the SOAPdenovo, a novel short-read assembly method where locations of protein-coding sequences, tRNA genes, and rRNA genes were identified.

Then the functions of these sequences were annotated by using the Prokka, an online platform for rapid annotation of prokaryotic genomes that performs a series of the process automatically (http://www.vicbioinformatics.com/software.prokka.shtml). The whole genome sequence of the BA04 strain was also annotated under RAST (Rapid annotation using subsystem technology) online annotation server (http://rast.theseed.org/FIG/rast.cgi) [56]. This annotation service determines the tRNA, rRNA, and protein-coding genes. Apart from these it also predicts the metabolic pathway involved in the genome by comparing it with other annotated genomes through the KEGG (Kyoto Encyclopaedia of Genes and genome) pathway. The Island Viewer 4 was used to predict the virulence and resistance genes and their location in the genome. (http://www.pathogenomics.sfu.ca/islandviewer/results/PBvHBZk27SXKvY1oH92c64/). The CRISPR elements were identified under CRISPR finder online web server based on the CRISPR database [51].

The whole genome phylogenetic analysis was performed on GGDC (Genome to genome distance calculator) platform, which compares the distance between genomes with pairwise alignment between the query and the searched database sequences. It also gives high support values and insignificant subspecies conflicts thereby enabling genome-based species delineation analogous to the traditional DNA-DNA hybridization method.

### Amplification and cloning of *Cry* gene

To amplify the identified full-length *Cry* gene sequences primers have been synthesized. Both forward and reverse primers were used to amplify MH753362 (Fw-5”-ATGAAACCCAAAAATCAAAATAAGTG-3’; Rv-5’-CTAAATGTTACGCTCAATATTGAGTTG-3’) and MH753363.1 (Fw- 5’-ATGGAACCTTATGCTGTATTATC-3’; Rv-5’- TTAACTTTTTGACACTTGAATTAAGT-3’). PCR program was set up for 35 cycles with pre-denaturation at 95 °C for 5 min, denaturation at 95 °C for 1 min, annealing 1.5 min at 48 °C for sequence MH753362.1 and 52 °C for sequence MH753363.1, extension at 72 °C for 2 min and final extension at 72 °C for 10 min. The PCR-amplified products were analyzed on 1% Agarose gel. The products were purified and cloned into the pGEM-T easy cloning vector.

The two novel types of *Cry* genes sequences were also processed for 3D analysis using the online server Phyre2 to study the structural similarities with the other crystal proteins (http://www.sbg.bio.ic.ac.uk/~phyre2/html/page.cgi?id=index).

## Supplementary Information

**Additional file 1:** **Supplementary figures**

**Additional file 2:** **Supplementary table 1**

**Additional file 3:** **Supplementary table 2**

## Data Availability

The whole genome sequences have been submitted to the NCBI [Accession number PUWY00000000(PUWY01000001-PUWY01000094) with Bioproject number PRJNA436085].
