# Supplementary material for: Whole genome sequencing of a novel Bacillus thuringiensis isolated from Assam soil
Source: BMC Microbiol. 2023 Mar 31;23:91. doi: 10.1186/s12866-023-02821-0 (PMC10064770; doi:10.1186/s12866-023-02821-0)
Supplement: Supplementary file 1 — Additional file 1: Supplementary figures [file 12866_2023_2821_MOESM1_ESM.docx]

Additional Figure S 1 : Representation of molecular interaction, reaction, and relation networks in KEGG pathway map for the whole genome sequence of isolate BA04 through RAST annotation server.


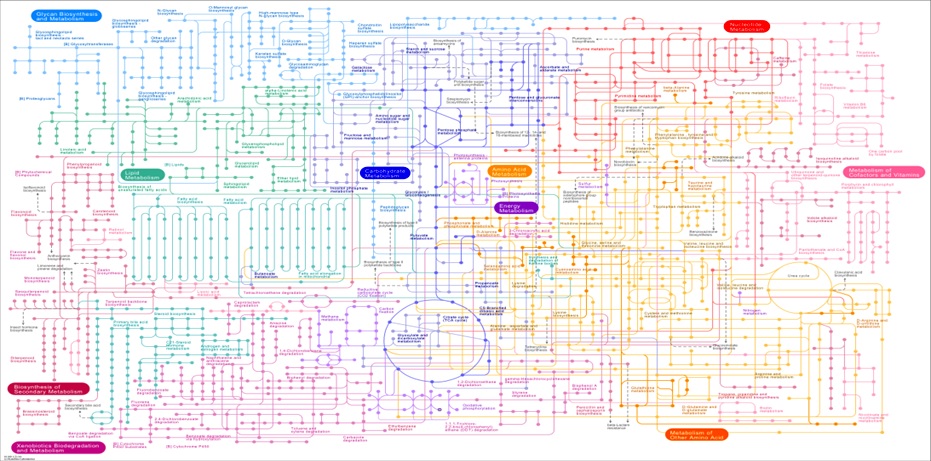


Additional Figure S2. Mortality of the Helicoverpa larvae on the artificial diet containing spores of *B. thuringiensis* strain BA04.


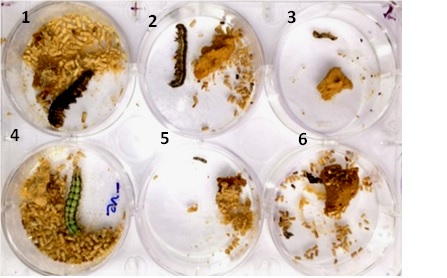


Insect on artificial diet having spores of BA04 1: 4^th^ instar larvae , 2: 3^rd^ instar larvae, 3: 2^nd^ instar larvae, 5: 1^st^ instar larvae, 6: Neonate after hatching.

4: 3^rd^ instar larvae on diet with out spores of BA04


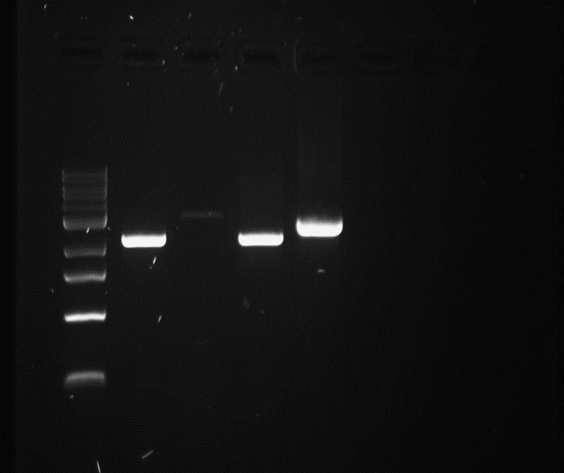


**Original Figure 6**: Amplification of full-length *Cry* gene of isolate BA04. Amplification was done using two different primer sets for the same gene. Lane M- 1kb DNA ladder, Lane 01- Amplified product of MH753362.1 using Primer 1A, Lane 02- Amplified product of MH753363.1 Primer 1B, Lane 01- Amplified product of MH753362.1 using Primer 1A.1, Lane 02- Amplified product of MH753363.1 using 1B.1
